# Dementia Risk in Type 2 Diabetes Patients: Acarbose Use and Its Joint Effects with Metformin and Pioglitazone

**DOI:** 10.14336/AD.2019.0621

**Published:** 2019-06-21

**Authors:** Chin-Hsiao Tseng

**Affiliations:** ^1^Department of Internal Medicine, National Taiwan University College of Medicine, Taipei, Taiwan; ^2^Division of Endocrinology and Metabolism, Department of Internal Medicine, National Taiwan University Hospital, Taipei, Taiwan; ^3^Division of Environmental Health and Occupational Medicine of the National Health Research Institutes, Zhunan, Taiwan

**Keywords:** acarbose, dementia, diabetes mellitus, metformin, pioglitazone, Taiwan

## Abstract

This population-based retrospective cohort study investigated dementia risk associated with acarbose in patients with type 2 diabetes mellitus by using Taiwan’s National Health Insurance database. A cohort of 15,524 matched pairs of ever and never users of acarbose based on propensity score matching was enrolled from new-onset type 2 diabetes patients from 1999 to 2006. Patients who were alive on January 1, 2007, were followed up for dementia until December 31, 2011. Adjusted hazard ratios were estimated using Cox proportional hazards models. The results revealed that the incident case numbers (incidence rates) of dementia were 264 (407.19 per 100,000 person-years) for never users and 231 (337.94 per 100,000 person-years) for ever users. The hazard ratio for ever users versus never users was 0.841 (95% confidence interval, 0.704-1.005) and 0.918 (0.845-0.998) for every 1-year increment of cumulative duration of acarbose therapy. Subgroup analyses showed that the reduced risk associated with acarbose was only observed in women (adjusted hazard ratio, 0.783; 95% confidence interval, 0.618-0.992) and in non-users of metformin (adjusted hazard ratio, 0.635; 95% confidence interval, 0.481-0.837). A model comparing different combinations of acarbose, metformin, and pioglitazone suggested that users of all three drugs had the lowest risk of dementia (hazard ratio, 0.406; 95% confidence interval, 0.178-0.925). In conclusion, reduced risk of dementia associated with acarbose is observed in the female sex and in non-users of metformin. Moreover, users of all three drugs (acarbose, metformin, and pioglitazone) have the lowest risk of dementia.

Dementia can either have a vascular etiology or occur because of a neurodegenerative disease such as Alzheimer’s disease (AD). Diabetes patients have a significantly 5-fold increased risk of dementia [1]. The close association between type 2 diabetes mellitus and AD and their potential common pathophysiological changes of impaired insulin expression and insulin resistance led to the coining of the term “type 3 diabetes” for AD [2]. The increased risk of dementia in diabetes patients may be due to the increased incidence of atherosclerosis, blood-brain barrier disturbances, and neurodegeneration associated with diabetes mellitus. The pathophysiological changes may include insulin resistance, increased deposition of advanced glycation end products, dysregulation of lipid metabolism, and augmented inflammation and oxidative stress [1,3]. Studies also suggest that postprandial glucose and glucose variability may increase the risk of cognitive dysfunction and dementia [4,5].

Major brain pathological changes of AD include deposition of amyloid beta (Aβ) and hyper-phosphorylation of tau protein [2]. Aβ is formed by the cleaving of the amyloid precursor protein by secretases [6], and insulin resistance in the brain may aggravate the accumulation of Aβ [7]. Additionally, AD is characterized by neurodegeneration with damage in cholinergic neurons, resulting in reduced release of acetylcholine neurotransmitters [8]. Acetylcholinesterase and butyryl-cholinesterase are serine hydrolases that are responsible for the catalytic hydrolysis of acetylcholine and they play an important role in the aggregation of Aβ [9]. Therefore, cholinesterase inhibitors are the main drugs currently approved for AD treatment [8,10].

Theoretically, antidiabetic drugs that improve insulin resistance in the brain can potentially prevent AD or dementia [2]. As shown in our previous observational studies, two antidiabetic drugs, specifically metformin [11] and pioglitazone [12], that improve insulin resistance, show a reduced risk of dementia in a dose-response pattern in patients with type 2 diabetes mellitus.

Acarbose, an alpha-glucosidase inhibitor that inhibits the digestion of carbohydrate in the intestine, is commonly used to treat diabetes in Asian populations, probably because of its glucose lowering effect for patients who consume Asian diets that have a high content of carbohydrate [13,14]. Acarbose has the following benefits that may contribute to a reduction of dementia risk: lowering postprandial glucose with a lower risk of hypoglycemia, improving insulin resistance, improving lipid profile, enhancing the release of glucagon-like peptide-1, inhibiting platelet activation, exerting anti-inflammatory effect, and reducing oxidative stress [13,15]. Indeed, novel drugs that may exert inhibitory effects on alpha glucosidase and cholinesterase are being developed for the treatment of both type 2 diabetes mellitus and AD [16].

A recent animal study suggested that acarbose has a protective effect on the decline of cognitive function, including spatial learning and memory, in SAMP8 mice [17]. However, a recent small scale randomized clinical trial conducted in patients with non-dementia vascular cognitive impairment and abnormal glucose metabolism showed an improvement in cognitive function only in patients assigned to metformin and donepezil (n = 50) for one year but not in those assigned to acarbose and donepezil (n = 50) [18]. Whether prolonged use of acarbose in diabetes treatment might exert a potential benefit for dementia has not been investigated. The present study investigated dementia risk in patients with type 2 diabetes mellitus who had been treated with acarbose and those who had never been treated with acarbose in the Chinese population in Taiwan by using the reimbursement database of the National Health Insurance (NHI).

## MATERIALS AND METHODS

This retrospective cohort study used the longitudinal reimbursement database of Taiwan’s NHI. The NHI is a unique healthcare system that covers more than 99.6% of Taiwan’s population; it has been implemented since March 1995. A majority of medical institutions throughout the nation (93%) have been contracted to the Bureau of NHI. The database keeps records of all disease diagnoses, medication prescriptions, and performed procedures. This can be used for academic research after ethics review.

The approval number for the present study was 99274 by the National Health Research Institutes and the database was described in detail in previous papers [19,20]. Throughout the study period, diabetes was coded 250.XX according to the International Classification of Diseases, Ninth Revision, Clinical Modification (ICD-9-CM) and dementia was coded as abridged codes of A210 or A222, or as ICD-9-CM codes of 290.0, 290.1, 290.2, 290.4, 294.1, 331.0-331.2, or 331.7-331.9.

**Figure 1.**
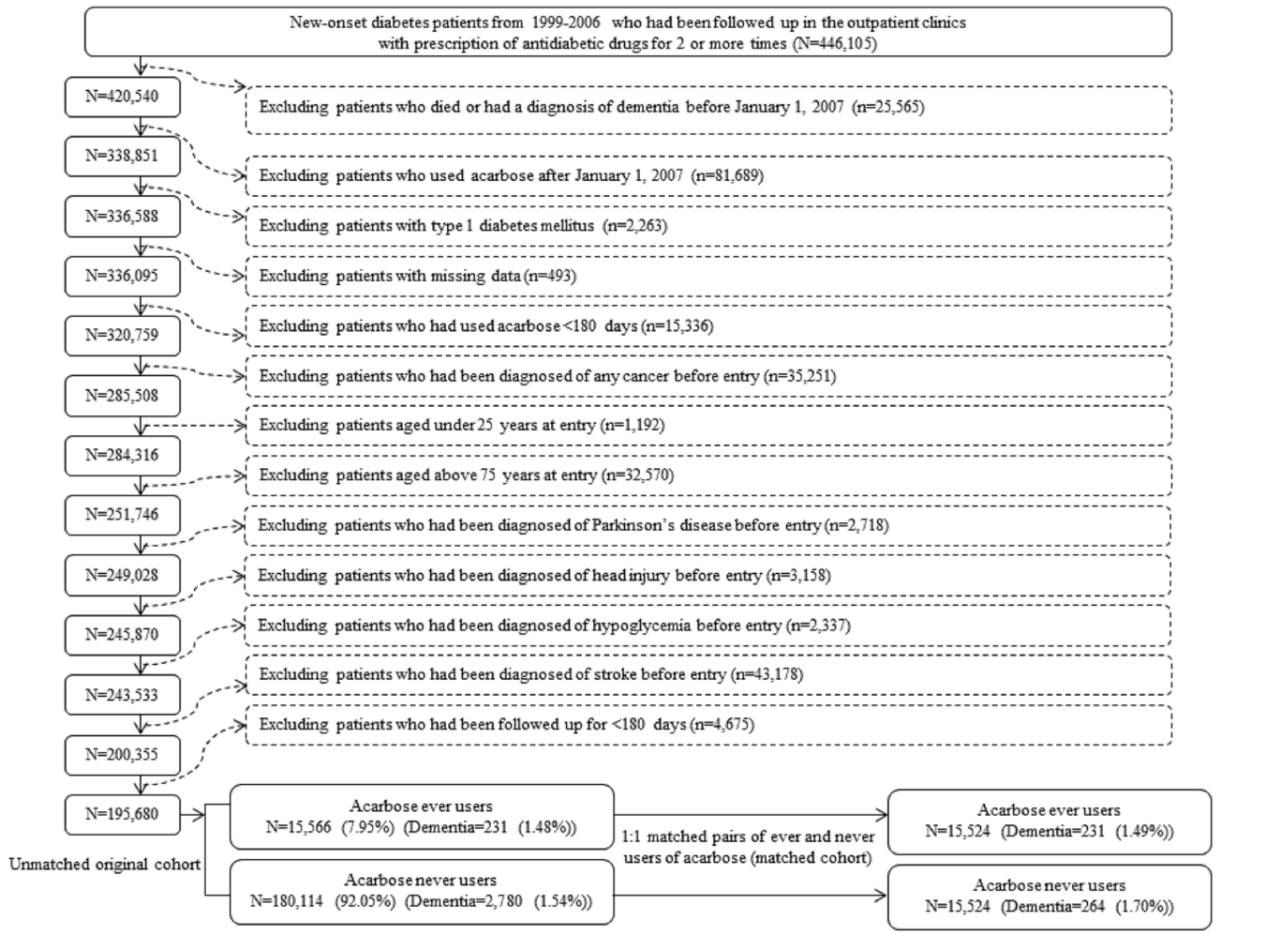
Flowchart for the procedures in selecting a propensity score-matched cohort of acarbose ever users and never users.

The procedures used to create a cohort of 1:1 matched pair of ever users and never users of acarbose are shown in Figure 1. At first, 446,105 patients who had new-onset diabetes mellitus from 1999 to 2006 and who had been prescribed antidiabetic drugs two or more times were identified from the outpatient clinics. Patients diagnosed with diabetes mellitus between 1996 and 1998 were excluded to ensure that only patients with new-onset diabetes were included in the study. The following patients were then excluded: 1) patients who had been diagnosed with dementia and/or died before January 1, 2007 (n = 25,565); 2) patients who were initiated with acarbose after January 1, 2007 (n = 81,689); 3) patients with type 1 diabetes mellitus (n = 2263); 4) patients with missing data (n = 493); 5) patients who had used acarbose for <180 days (n = 15,336); 6) patients with a diagnosis of any cancer before entry (n = 35,251, cancer patients were excluded because they might have shortened lifespans which might have distorted follow-up time and dementia could be misdiagnosed from the clinical presentations of malignancy); 7) patients aged <25 years (n = 1192); 8) patients aged >75 years (n = 32,570); 9) patients with Parkinson’s disease before entry (n = 2718); 10) patients with head injury before entry (n = 3158); 11) patients with a diagnosis of hypoglycemia before entry (n = 2337); 12) patients with a history of stroke before entry (n = 43,178); and 13) patients with a follow-up duration < 180 days (n = 4675). Patients with Parkinson’s disease, head injury, hypoglycemia, and stroke were excluded because these diagnoses are important risk factors for dementia. As a result, 15,566 ever users and 180,114 never users of acarbose were identified (unmatched original cohort). A cohort of 1:1 matched pairs of 15,524 ever users and 15,524 never users (the matched cohort) was created by propensity score matching based on the Greedy 8→1 digit match algorithm [21]. Logistic regression was used to create the propensity score from all characteristics listed in Table 1 being treated as independent variables.

The cumulative duration of acarbose therapy in years was calculated from the database. Potential confounders included the following categories: demographic data, major comorbidities associated with diabetes mellitus, diabetes-related complications, potential risk factors for cancer, potential factors that may affect the prescription of acarbose, antidiabetic drugs, and medications commonly used in diabetes patients. Demographic data included age, sex, diabetes duration, occupation, and living region (classified as Taipei, Northern, Central, Southern, and Kao-Ping/Eastern). Occupation was classified as class I (civil servants, teachers, government employees or private businesses, professionals, and technicians), class II (people without a specific employer, self-employed people, or seamen), class III (farmers or fishermen) and class IV (low-income families supported by social welfare, or veterans). The ICD-9-CM codes for potential confounders categorized as major comorbidities associated with diabetes mellitus (hypertension, dyslipi-demia, and obesity), diabetes-related complications (nephropathy, eye disease, ischemic heart disease, and peripheral arterial disease), potential risk factors for cancer (chronic obstructive pulmonary disease, tobacco abuse, and alcohol-related diagnoses), and potential factors that may affect the prescription of acarbose (gallstone, diseases of the digestive system, Helicobacter Pylori infection, and/or Helicobacter Pylori eradication therapies, hepatitis B virus infection, hepatitis C virus infection, liver cirrhosis, and other chronic non-alcoholic liver diseases) can be found in previously published papers [11,12,22]. Antidiabetic drugs included insulin, sulfonylurea, metformin, meglitinide, rosiglitazone, and pioglitazone. Commonly used medications in diabetes patients included angiotensin-converting enzyme inhibitors/angiotensin receptor blockers, calcium channel blockers, statins, fibrates, and aspirin.

Student’s t test was used to compare the differences of age and diabetes duration between never and ever users and the Chi-square test was used for other variables. According to Austin and Stuart, a standardized difference was calculated for each covariate and a cutoff value of >10% was used as an indicator of potential confounding from the variable [23].

Because previous animal studies showed that acarbose may exert a better anti-aging effect in male mice than in female mice [24] and the use of either metformin [11] or pioglitazone [12] may reduce the risk of dementia, the incidence density of dementia was calculated for ever users and never users of acarbose in all patients and in subgroups of men, women, patients with metformin use, patients without metformin use, patients with pioglitazone use and patients without pioglitazone use, respectively, and for different subgroups of combinations of the use of acarbose, metformin and pioglitazone. The numerator was the number of newly diagnosed cases of dementia patients identified during follow-up and the denominator was the follow-up duration in person-years. Follow-up started on January 1, 2007, and ended on December 31, 2011, at the time of a new diagnosis of dementia, or on the date of death or the last reimbursement record, whichever occurred first.

Cox proportional hazards model was used to estimate the adjusted hazard ratios and their 95% confidence intervals for ever users versus never users of acarbose, and for the cumulative duration (every 1-year increment) of acarbose therapy being treated as a continuous variable in all patients, and in men and women, respectively. To evaluate the effects of acarbose in the presence or absence of metformin or pioglitazone, hazard ratios for acarbose ever users versus never users were estimated in subgroups of patients with metformin use, without metformin use, with pioglitazone use and without pioglitazone use, respectively. Finally, the joint effects of the use of acarbose, metformin, and pioglitazone were evaluated in a model that estimated the hazard ratios in different subgroups of all possible combinations of the use of the three drugs by using a referent group including patients without the use of any of the three drugs. All baseline characteristics were considered as potential confounders and were adjusted for in the aforementioned Cox proportional hazards models.

Kaplan-Meier curves for dementia-free probability were plotted for acarbose never users versus ever users in all patients, in men, in women and in different subgroups of all possible combinations of the use of acarbose, metformin, and pioglitazone. Logrank test was used to test the significance among different subgroups of acarbose exposure.

Analyses were conducted using SAS Version 9.4 (SAS Institute, Cary, NC). P-value < 0.05 was considered statistically significant.

**Table 1:** Characteristics in never and ever users of acarbose.

Variable	Never users		Ever users			
	(n=15524)		(n=15524)		*P-*value	Standardized difference
	n	%	n	%		
Demographic data						
Age (years)	59.76	10.10	59.75	9.95	0.9225	-0.13
Sex (men)	8358	53.84	8443	54.39	0.3330	1.15
Diabetes duration (years)	5.42	2.41	5.45	2.27	0.2444	1.26
Occupation						
I	6749	43.47	6782	43.69	0.6102	
II	3687	23.75	3609	23.25		-1.24
III	2515	16.20	2580	16.62		1.14
IV	2573	16.57	2553	16.45		-0.34
Living region						
Taipei	5923	38.15	5884	37.90	0.6517	
Northern	1859	11.98	1785	11.50		-1.55
Central	1859	11.98	2736	17.62		0.80
Southern	1962	12.64	1990	12.82		0.61
Kao-Ping and Eastern	3089	19.90	3129	20.16		0.70
Major comorbidities						
Hypertension	10796	69.54	10777	69.42	0.8149	-0.34
Dyslipidemia	11515	74.18	11498	74.07	0.8256	-0.35
Obesity	1036	6.67	959	6.18	0.0747	-2.08
Diabetes-related complications						
Nephropathy	2717	17.50	2729	17.58	0.8579	0.23
Eye disease	3864	24.89	3861	24.87	0.9686	0.01
Ischemic heart disease	5253	33.84	5234	33.72	0.8197	-0.34
Peripheral arterial disease	2642	17.02	2614	16.84	0.6718	-0.51
Potential risk factors of cancer						
Chronic obstructive pulmonary disease	5657	36.44	5597	36.05	0.4787	-0.84
Tobacco abuse	385	2.48	385	2.48	0.3738	-1.07
Alcohol-related diagnoses	747	4.81	734	4.73	0.7292	-0.45
Potential factors that may affect the prescription of acarbose						
Gallstone	1335	8.60	1293	8.33	0.3918	-1.06
Diseases of the digestive system	15332	98.76	15337	98.80	0.7961	0.25
Helicobacter Pylori infection and/or Helicobacter Pylori eradication therapies	2904	18.71	2888	18.60	0.8157	-0.36
Hepatitis B virus infection	355	2.29	352	2.27	0.9091	-0.24
Hepatitis C virus infection	560	3.61	553	3.56	0.8308	-0.37
Liver cirrhosis	421	2.71	492	3.17	0.0171	2.64
Other chronic non-alcoholic liver diseases	1482	9.55	1508	9.71	0.6169	0.59
Antidiabetic drugs						
Insulin	740	4.77	781	5.03	0.2810	1.10
Sulfonylurea	10745	69.22	10845	69.86	0.2175	1.19
Metformin	9374	60.38	9370	60.36	0.9630	-0.48
Meglitinide	1114	7.18	1104	7.11	0.8256	-0.27
Rosiglitazone	1381	8.90	1407	9.06	0.6058	0.54
Pioglitazone	1397	9.00	1415	9.11	0.7219	0.34
Medications commonly used in diabetes patients				
Angiotensin converting enzyme inhibitor/angiotensin receptor blocker	9604	61.87	9570	61.65	0.6913	-0.55
Calcium channel blocker	7278	46.88	7262	46.78	0.8556	-0.24
Statin	8970	57.78	8810	56.75	0.0664	-2.12
Fibrate	5823	37.51	5870	37.81	0.5820	0.59
Aspirin	7065	45.51	7050	45.41	0.8643	-0.30

Age and diabetes duration are shown as mean and standard deviation.

### RESULTS

Table 1 shows the characteristics of never users and ever users of acarbose. None of the calculated values of standardized difference between the two groups was found to be > 10%, suggesting that the two groups were well matched in these covariates. However, the proportion of liver cirrhosis in ever users was slightly higher than that in never users (3.17% versus 2.71%, P-value = 0.0171).

The incidence rates of dementia and the adjusted hazard ratios by acarbose exposure in all patients and in different sexes are shown in Table 2. In all patients, the adjusted hazard ratio comparing acarbose ever users versus never users suggested a non-significant risk reduction associated with acarbose use (adjusted hazard ratio, 0.841; 95% confidence interval, 0.704-1.005). When treated as a continuous variable, the cumulative duration of acarbose therapy was significantly associated with a reduced risk. In the analyses conducted in men and women separately, acarbose showed a neutral effect in men but a protective effect in women.

Table 3 shows the results of models analyzing the effects of acarbose on dementia risk with regard to exposure to metformin and/or pioglitazone. The findings suggested that the reduced risk of dementia associated with acarbose use was mainly observed in non-users of metformin (model II) and was not significantly observed in patients who were using metformin (model I). The use (model III) or non-use of pioglitazone (model IV) did not significantly affect the risk of dementia associated with acarbose. In the model evaluating the joint effects of all possible combinations of the use of acarbose, metformin, and pioglitazone (model V), patients who were using all the three drugs (group 7) showed a significantly low risk of dementia. Patients who were using one or two of the three drugs showed either a non-significant association (groups 2, 3. and 6) or a significant risk reduction (groups 1, 4, and 5).

**Table 2:** Incidence rates of dementia and hazard ratios by acarbose exposure in all patients and in different sexes.

Models	*n*	*N*	Person-years	Incidence rate (per 100,000 person-years)	Adjusted hazard ratio	95% Confidence interval	*P*-value
All patients							
Acarbose never users	264	15524	64834.36	407.19	1.000		
Acarbose ever users	231	15524	68355.38	337.94	0.841	(0.704-1.005)	0.0561
Cumulative duration of acarbose therapy treated as a continuous variable							
For every 1-year increment of acarbose use					0.918	(0.845-0.998)	0.0444
Men							
Acarbose never users	101	8358	34603.53	291.88	1.000		
Acarbose ever users	107	8443	37111.21	288.32	0.934	(0.710-1.228)	0.6252
Cumulative duration of acarbose therapy treated as a continuous variable							
For every 1-year increment of acarbose use					0.983	(0.871-1.110)	0.7840
Women							
Acarbose never users	163	7166	30230.84	539.18	1.000		
Acarbose ever users	124	7081	31244.17	396.87	0.783	(0.618-0.992)	0.0425
Cumulative duration of acarbose therapy treated as a continuous variable							
For every 1-year increment of acarbose use					0.870	(0.775-0.976)	0.0180

*n*: incident cases of dementia, *N*: cases followed

**Figure 2.**
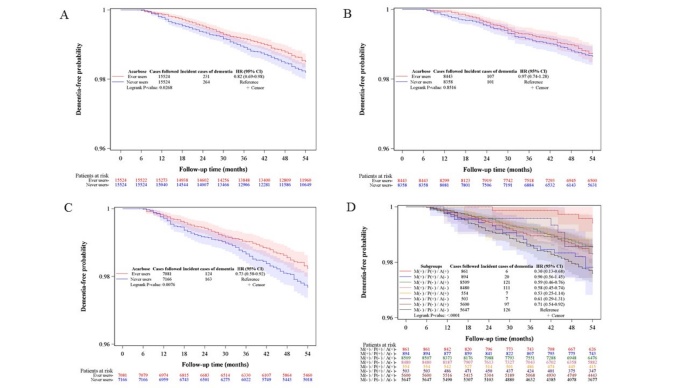
Kaplan-Meier curves comparing dementia-free probability in different subgroups of acarbose exposure. Kaplan-Meier curves comparing dementia-free probability between ever users and never users of acarbose in both sexes together (A) and in separate sexes (B: men, C: women). (D) compares the respective curves in subgroups of patients with different combinations of use (+) and non-use (-) of metformin (M), pioglitazone (P) and acarbose (A). The 95% confidence intervals are shown in shaded areas. HR: hazard ratio; CI: confidence interval.

Kaplan-Meier curves comparing dementia-free probability in different subgroups of acarbose exposure are shown in Figure 2. Figure 2A, 2B and 2C compare ever and never users of acarbose in all patients (logrank P-value = 0.0268), in men (logrank P-value = 0.8516), and in women (logrank P-value = 0.0076), respectively. Figure 2D compares the dementia-free probability among the subgroups of different combinations of the use of acarbose, metformin, and pioglitazone. The logrank test (P-value < 0.0001) suggested a significant difference among the subgroups and patients who were using all three drugs had the lowest risk of developing dementia.

### DISCUSSION

The findings suggested that acarbose use in patients with type 2 diabetes mellitus might exert some protective effect against dementia (Table 2), and that such a benefit was mainly observed in female patients (Table 2 and Figure 2C) and in non-users of metformin (model II of Table 3). In the analyses of the joint effects of acarbose, metformin and pioglitazone, the lowest risk of dementia was observed in those who were using all three drugs (group 7 in model V of Table 3 and Figure 2D).

As shown in our previous studies, metformin [11] and pioglitazone [12] improve insulin resistance and are associated with a lower risk of dementia. This study provided information to support the potential role of insulin resistance in dementia in patients with type 2 diabetes mellitus because acarbose may also reduce insulin resistance, though to a lesser extent. However, unlike metformin and pioglitazone that may pass through the blood-brain barrier [11,12], acarbose or its metabolites do not cross the blood-brain barrier [24]. Therefore, acarbose may not act directly on the central nervous system. Acarbose is especially useful for reducing postprandial glucose and it is less likely to cause hypoglycemia [13,15]. Therefore, the reduced oxidative stress because of reduced glucose variability in patients treated with acarbose may contribute to a reduced risk of dementia. The improvement of lipid profile, inhibition of platelet activation, and the reduction of inflammation associated with acarbose use [13,15] may also partly explain the reduced risk of dementia associated with acarbose.

The subgroup analyses suggested that the beneficial effect of acarbose on dementia was mainly observed in female patients (Table 2) and in non-users of metformin (model II of Table 3). Metformin is protective against dementia per se [11] and it is always used as a first-line treatment for type 2 diabetes mellitus. Therefore, patients who used both metformin and acarbose in the study might have been benefited from the protection against dementia by metformin and the benefit of acarbose might not be additive to the effect of metformin. It is worthy to note that the beneficial effect of metformin on dementia could also be demonstrated in the present study by comparing the incidence rates of dementia in patients who were using metformin versus those who were not using metformin in never users of acarbose (332.42 versus 523.07 per 100,000 person-years) and in ever users of acarbose (309.78 versus 380.13 per 100,000 person-years), respectively (Models I and II of Table 3). Similarly, female patients seemed to suffer from a higher incidence of dementia than male patients in acarbose never users and ever users, respectively (Table 2).

The differential effects of acarbose on dementia risk in male and female patients are interesting. Previous animal studies also suggested differential effects of acarbose on lifespan prolongation in male and female mice [24]. While acarbose increased the median lifespan by 22% in male mice (P-value < 0.0001), it increased the lifespan in female mice by only 5% (P-value = 0.01) [24]. Although acarbose was beneficial in prolonging the lifespan in male mice [24], its benefit on dementia seemed to be more predominant in the female patients in the present study (Table 2). The reason for such a discrepancy between different sexes in terms of lifespan in mice and in terms of dementia in humans remains unknown.

**Table 3:** The effects of acarbose on dementia risk with regards to exposure to metformin and/or pioglitazone.

Models	*n*	*N*	Person-years	Incidence rate (per 100,000 person-years)	Adjusted hazard ratio	95% Confidence interval	*P*-value
1.Patients with metformin							
Acarbose never users	131	9374	39407.59	332.42	1.000		
Acarbose ever users	127	9370	40996.20	309.78	1.023	(0.798-1.312)	0.8559
2.Patients without metformin							
Acarbose never users	133	6150	25426.78	523.07	1.000		
Acarbose ever users	104	6154	27359.18	380.13	0.635	(0.481-0.837)	0.0013
3.Patients with pioglitazone							
Acarbose never users	27	1397	6101.85	442.49	1.000		
Acarbose ever users	13	1415	6098.01	213.18	0.598	(0.303-1.180)	0.1382
4.Patients without pioglitazone							
Acarbose never users	237	14127	58732.51	403.52	1.000		
Acarbose ever users	218	14109	62257.37	350.16	0.877	(0.728-1.055)	0.1644
5.Joint effects of metformin, pioglitazone and acarbose					
Group 0: Metformin (-) / Pioglitazone (-) / Acarbose (-)	126	5647	23323.77	540.22	1.000		
Group 1: Metformin (-) / Pioglitazone (-) / Acarbose (+)	97	5600	24953.18	388.73	0.643	(0.489-0.845)	0.0015
Group 2: Metformin (-) / Pioglitazone (+) / Acarbose (-)	7	503	2103.01	332.86	0.635	(0.295-1.369)	0.2466
Group 3: Metformin (-) / Pioglitazone (+) / Acarbose (+)	7	554	2405.99	290.94	0.603	(0.281-1.295)	0.1945
Group 4: Metformin (+) / Pioglitazone (-) / Acarbose (-)	111	8480	35408.74	313.48	0.596	(0.457-0.779)	0.0002
Group 5: Metformin (+) / Pioglitazone (-) / Acarbose (+)	121	8509	37304.19	324.36	0.691	(0.535-0.893)	0.0048
Group 6: Metformin (+) / Pioglitazone (+) / Acarbose (-)	20	894	3998.85	500.14	1.104	(0.683-1.784)	0.6870
Group 7: Metformin (+) / Pioglitazone (+) / Acarbose (+)	6	861	3692.01	162.51	0.406	(0.178-0.925)	0.0320

*n*: incident cases of dementia, *N*: cases followed

In recent decades, there has been a dramatic increase in diabetes worldwide, and millions of patients are using antidiabetic drugs. This study may have some clinical and research significance because acarbose remains an important oral antidiabetic drug, especially in Asian populations. Although no definite beneficial effects on cardiovascular disease were observed with acarbose treatment in a clinical trial conducted in the Chinese population in China [25], the potential benefit of acarbose on dementia in specific subgroups of patients, i.e., in female patients (Table 2), in non-users of metformin (model II of Table 3) and in combination with metformin and pioglitazone (group 7 in model V of Table 3), provides rationale for repositioning acarbose in the treatment of Chinese patients with type 2 diabetes mellitus. At least, based on current evidence, acarbose may be considered in patients who consume large amounts of carbohydrate and it can be used as a substitute for metformin in patients who are at risk of dementia but contraindicated for metformin use or who cannot tolerate the side effects of metformin, especially in female patients. A combination of acarbose, metformin, and pioglitazone may be a good choice in some patients who require greater control of blood glucose. Such a combination theoretically provides a better glycemic control without the risk of hypoglycemia and with a much more reduced insulin resistance. The findings of the present study also provide rationale for the development of novel drugs that can pass through the blood-brain barrier and exert dual inhibitory effects on alpha glucosidase and cholinesterase for targeting the treatment of both type 2 diabetes mellitus and AD [16].

The present study has considered some potential biases commonly encountered in pharmaco-epidemiological studies that use pre-existing administrative databases. These biases include selection bias, prevalent user bias, immortal time bias, and confounding by indication. By using the nationwide database that covers more than 99.6% of the population, selection bias is not a problem. Because the patients should have had new-onset type 2 diabetes mellitus during the enrollment period and the use of acarbose was counted from the longitudinal database since its very first prescription, we included incident users of acarbose and avoided the potential risk of prevalent user bias.

Immortal time refers to the follow-up period when the outcome cannot occur. Therefore, immortal time bias can be introduced when the treatment status or follow-up time is inappropriately assigned [26]. In the present study, ambiguous diagnosis of diabetes was unlikely because only patients who had been prescribed antidiabetic drugs two or more times had been enrolled (Figure 1). Misclassification of treatment status was also unlikely in Taiwan because the NHI is a universal healthcare system and the information of all prescriptions was completely stored in computer files during the whole study period. Therefore, misdiagnosis of diabetes and misclassification of treatment status were well avoided.

The follow-up time of the patients was also carefully considered to avoid the inclusion of immortal time. First, immortal time bias was avoided by excluding patients who had a short follow-up period of < 180 days (Figure 1). The immortal time between diabetes diagnosis and the start of the use of antidiabetic drugs had been avoided because patients were enrolled only when they started with the treatment of antidiabetic drugs and immortal time was actually not calculated in the person-years. The immortal time introduced during the waiting period between the prescription and the dispensing of medications after hospital discharge as described by Lévesque et al. [26] would not occur in Taiwan because all discharge medications can be obtained directly from the hospital when the patients are discharged.

Confounding by indication could be reduced by using a cohort of propensity score-matched pairs of ever and never users of acarbose (Figure 1). Because none of the covariates had a standardized difference > 10% in the matched cohort (Table 1), residual confounding by indication was not likely. It was noted that the distribution of liver cirrhosis differed significantly between ever users and never users of acarbose (Table 1). However, this would probably not exert a confounding effect because the difference was small and such an effect has been adjusted for in the estimation of the hazard ratios (Tables 2 and 3).

Some other merits deserve mentioning. Because the NHI database covers > 99.6% of Taiwan’s population, the findings can be readily generalized to the whole population. The potential bias resulting from self-reporting could be further reduced by using medical records. Detection bias resulting from different socioeconomic statuses is less likely in our healthcare system because the drug cost sharing is low and can always be waived off for low-income patients, veterans, and prescription refills for chronic diseases.

The study limitations may include a lack of records of blood levels of glucose and insulin, a lack of glucose excursion index, a lack of indicators of insulin resistance, and β-cell function for more in-depth analyses. Furthermore, the information of some confounders like anthropometric factors, dietary pattern, nutritional status, lifestyle, smoking, alcohol drinking, family history, and genetic parameters was not available. Because only approximately 9% of the enrolled patients were pioglitazone users (Table 1), the models evaluating the dementia risk in subgroups of patients with pioglitazone use had low numbers of incident cases (models III and V of Table 3); therefore, the impact of these models might not be sufficient.

In conclusion, the present study finds a potential benefit of acarbose in reducing the risk of dementia in Taiwanese patients with type 2 diabetes mellitus. This benefit is especially significant in female patients, in non-users of metformin, and in patients who are treated with a combination of acarbose, metformin, and pioglitazone.
